# Four weeks of supervised home-based aerobic cycling improves cardiopulmonary function in patients with Parkinson’s disease

**DOI:** 10.3389/fneur.2026.1761398

**Published:** 2026-03-13

**Authors:** Yang Jiao, Guang Xin Liu, Jin Wang, Yue Wang, Weijia Hou, Yang Yu, Zhizhong Zhu, Mei Ma

**Affiliations:** 1 Department of Rehabilitation Medicine, Huanhu Hospital Affiliated to Tianjin Medical University, Tianjin, China; 2 Department of Rehabilitation Medicine, Tianjin Chest Hospital, Tianjin, China; 3 Department of Neurology, Huanhu Hospital Affiliated to Tianjin Medical University, Tianjin, China; 4 Department of Rehabilitation, Tianjin University Huanhu Hospital, Tianjin, China; 5 Tianjin Key Laboratory of Cerebral Vascular and Neurodegenerative Diseases, Tianjin, China

**Keywords:** aerobic cycling, cardiopulmonary function, home-based exercise, Parkinson’s disease, rehabilitation interventions

## Abstract

**Objective:**

To investigate the effects of a short-term, supervised home-based aerobic cycling intervention on cardiopulmonary function in patients with mild-to-moderate PD.

**Design:**

A single-center, pre-post intervention study was conducted. Seventeen PD patients (Hoehn-Yahr stage 1–3) underwent a 4-week supervised home-based cycling program, exercising at 70–80% of heart rate reserve for 40 min per session, three times per week. Remote supervision was facilitated via a mobile application. The primary outcome was maximal power output (W). Secondary outcomes included anaerobic threshold (AT), peak oxygen uptake (VO₂peak), heart rate parameters, and pulmonary function tests. Assessments were performed at baseline and after the intervention in the ON medication state.

**Results:**

After the 4-week intervention, key cardiopulmonary parameters showed significant increases in Maximal power output (*p* < 0.001) and peak oxygen uptake (VO₂peak) (*p* = 0.006). Anaerobic threshold power and related heart rate measures also showed significant enhancement (*p* < 0.05). Pulmonary function parameter revealed a slight improvement in peak expiatory flow (*p* < 0.05).

**Conclusion:**

A 4-week supervised home-based aerobic cycling program is a feasible and effective intervention, leading to significant improvements in aerobic capacity, metabolic efficiency, and cardiovascular response in patients with mild-to-moderate PD.

## Introduction

Parkinson’s disease (PD) is a progressive neurodegenerative disorder classically characterized by motor symptoms including bradykinesia, tremor, and rigidity (1). However, accumulating evidence increasingly emphasizes the pivotal role of non-motor manifestations—particularly cardiopulmonary dysfunction—in shaping disease prognosis and patients’ quality of life. Previous study confirms that cardiopulmonary exercise testing (CPET) is safe and feasible for PD patients (2). Significantly, research has revealed a robust inverse correlation between VO₂max, peak power output, and disease severity (as measured by MDS-UPDRS scores) (3), indicating that the motor impairments of bradykinesia and akinesia in PD may hinder patients from achieving sufficient exercise intensity to reach VO₂peak, thereby restricting maximal cardiorespiratory demand. Consequently, individuals with PD often exhibit a deconditioned state. This interplay between motor and non-motor symptoms underscores the pressing need to target cardiopulmonary function in PD management—not only to alleviate disability but also to address a modifiable factor contributing to mortality risk.

The Cyclical Lower Extremity Exercise (CYCLE) trial has demonstrated that high-intensity aerobic cycling, whether voluntary or forced, significantly enhances motor function in patients with Parkinson’s disease (PD) (4). Neuroimaging findings have revealed that a single cycling session can strengthen functional connectivity in the motor cortex, generating neuromodulatory effects that mechanistically parallel those of dopaminergic medications (5, 6). Building on these insights, the present project aimed to analyze cardiopulmonary function-related data derived from cycling training, to determine the impact of an aerobic exercise intervention on cardiopulmonary responses in terms of aerobic capacity among individuals with PD.

Van der Kolk and colleagues found 24-week home-based cycling intervention demonstrated significant cardiopulmonary improvements in patients with Parkinson’s disease (PD), as evidenced by an increase in VO₂max of 2.0 mL/kg/min compared with a decrease of 0.4 mL/kg/min in the control group, resulting in a mean difference of 2.4 mL/kg/min (95% confidence interval [CI]: 1.1–3.7) (7). Although current clinical guidelines recommend personalized adjustments of exercise intensity and volume based on disease progression, long-term adherence remains a critical challenge, particularly in patients with fluctuating symptoms (8). To address this gap, we implemented a 4-week standardized cycling protocol targeting patients with mild-to-moderate PD (Hoehn-Yahr stage 1–3) using a self-controlled study design. Systematic assessments of parameters such as anaerobic threshold power, maximal power, and peak VO₂ will provide actionable data to inform short-term exercise prescription within an evidence-based framework.

## Methods

### Study design

**Figure 1:**
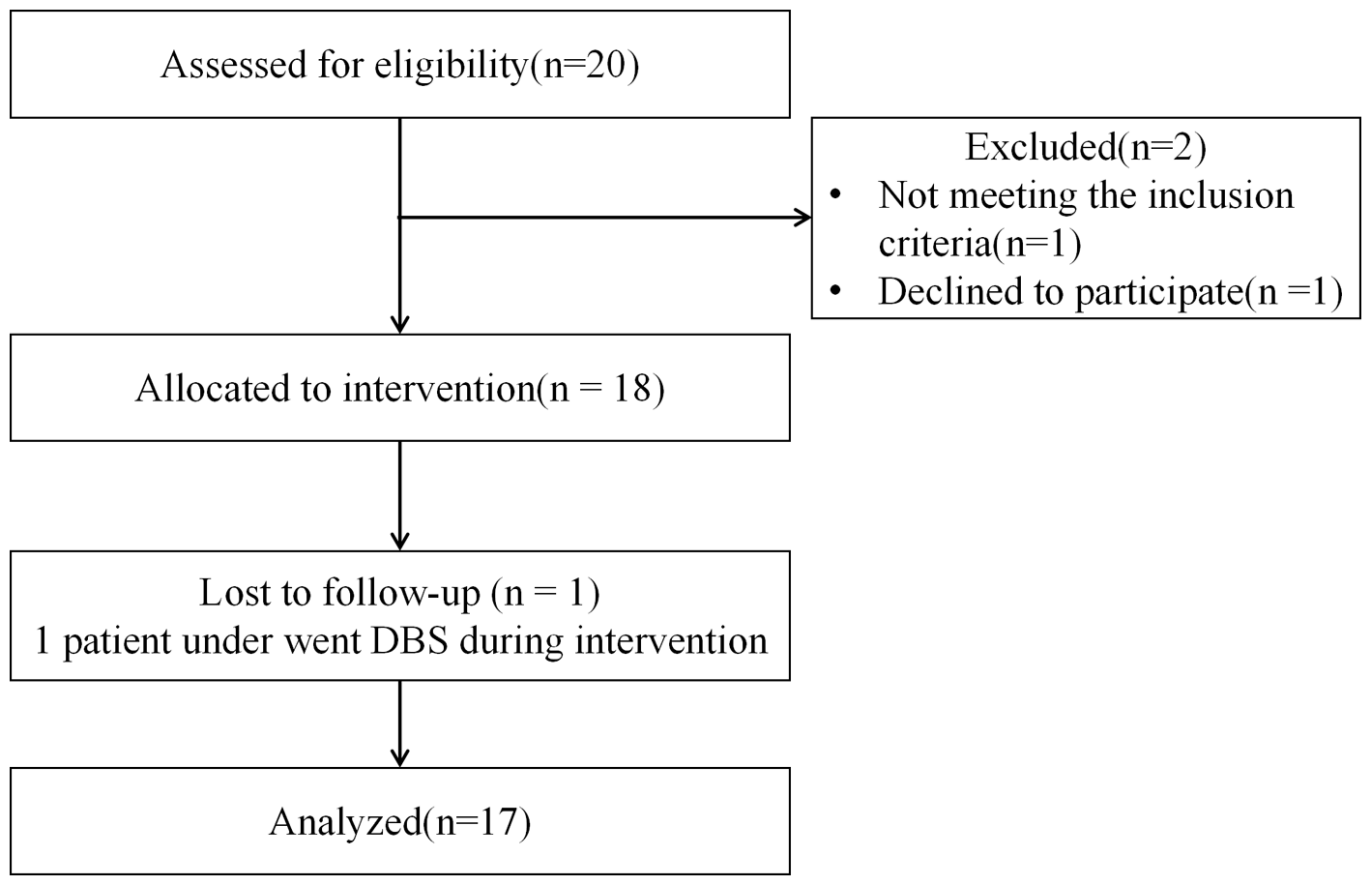
Flow diagram.

Study participants were recruited from the outpatient clinic of the Huanhu Hospital, Tianjin, China, to participate in a single-center, home-based, pre-post intervention study aimed at exploring the improvement effect of aerobic exercise on patients with Parkinson’s disease. Assessments were done at the Department of Rehabilitation Medicine. The intervention in participants was delivered in the patients’ homes, with remote supervision by a rehabilitation physician or physical therapist (see Figure 1).

The criteria for eligibility were as follows: Patients with clinically confirmed Parkinson’s disease (diagnosed per the Chinese PD Diagnostic Criteria [2016] and MDS criteria) were eligible if they exhibited gait impairment, verified by a movement disorders specialist through clinical evaluation, along with an MDS-UPDRS III-item 11 score >1, while maintaining a stable medication regimen without frequent dose adjustments.

Exclusion criteria were as follows: Patients with neurological, orthopedic, or cardiac comorbidities that make them unfit to do aerobic or stretching exercises; patients with psychiatric diseases diagnosed in the past year by a psychiatrist; patients with dementia, who were unable to complete questionnaires or finish the training program; patients over 75 years old or under 18 years old, and those with a history of Parkinson’s disease surgery (including: DBS and lesion surgery, etc.); and patients who were unavailable for more than 10% of the study period. Changes in medication during participation were discouraged but allowed at the discretion of the treating physician, creating a realistic, real-life clinical setting.

The sample size of this study was estimated based on the results of previous pilot studies and similar preliminary studies in the field of PD rehabilitation (7, 9). Taking peak oxygen uptake (VO₂peak) as the primary outcome indicator, with *α* = 0.05 and *β* = 0.2, the minimum required sample size was estimated to be 15 cases. Finally, 17 patients were included to cope with potential loss to follow-up and ensure statistical power.

This study was approved by the Ethics Committee of Tianjin Huanhu Hospital (No. 2023-124) and registered with the Chinese Clinical Trial Registry (ChiCTR2300074453). This study conforms to all STROBE guidelines and reports the required information accordingly (see Supplementary STROBE Checklist). All participants provided written informed consent prior to their involvement in the research.

### Intervention

All patients were trained 3 times per week for 4 weeks, for a total of 12 sessions at home under remote supervision. Remote supervision was implemented through a closed-loop management model of mobile application + WeChat workgroup: Patients wore HUAWEI Band 4 to collect real-time heart rate data, which was synchronized to the mobile application. Rehabilitation physicians/physical therapists could real-time monitor the exercise heart rate, duration, and power output to ensure the exercise intensity remained within the target range of 70–80% HRR. If there was an abnormal heart rate or exercise interruption, the physician would provide immediate guidance via WeChat. Within 24 h after each training session, the physician would review the data to make personalized program adjustments; a weekly online Q&A session was held to proactively follow up with patients who failed to complete training and identify the reasons, ensuring the standardization and adherence of the intervention.

### Outcome assessment

Before the first training session and after finishing the training program, all patients were tested in a standardized on state (1–1.5 h after taking medication). The patients were screened for cognitive function by the Mini-Mental State Examination. The severity of PD was evaluated by HY and the MDS-UPDRS III score. All participants underwent cardiopulmonary exercise testing (CPET) using a stationary cycle ergometer (RegoSelect 600, GE, United States) coupled with a cardiopulmonary exercise testing system (MasterScreen™ CPX, Jaeger, Germany). The entire testing protocol was supervised by a cardiologist.

Outcomes were assessed at baseline (TO) and after the 4 weeks, when the program was completed (T1). The CPET was done in a standardized on state. The primary outcome was maximal power output [W] at T0 and T1, measured in a standardized on state (as defined above). Secondary outcomes included the following parameters: resting heart rate (bpm), anaerobic threshold (AT)-related measures (power output [W], VO₂/kg [mL/kg/min], and heart rate [bpm]), peak exercise capacity metrics (peak VO₂/kg [mL/kg/min], and maximal heart rate [bpm]), metabolic indices (maximal fat oxidation rate [g/h] and fat-burning heart rate [bpm]), as well as ventilatory efficiency parameters (VE/VCO₂ slope and oxygen uptake efficiency slope [OUES]). And pulmonary function parameters:forced vital capacity (FVC, L), forced expiratory volume in 1 s (FEV₁, L), peak expiratory flow (PEF, L/s), and maximum voluntary ventilation (MVV, L/min).

Two trained and certified raters assessed all outcomes for a single patient. Patients were instructed to report adverse events directly to the physician. At the end of the T1 visit, adverse events were also reported retrospectively.

### Statistical analysis

All parameters were presented as group means ± standard deviations. The Shapiro–Wilk test was employed to assess the distributional characteristics of the parameters. For paired-sample comparisons (between the PRE and POST training sessions), the Wilcoxon signed-rank test or Student’s *t*-test was selected based on the results of the Shapiro–Wilk test and the type of scale applied, to examine the statistical significance of any existing differences. An *α* value ≤0.05 was taken as statistically significant for all analyses, which were performed using SPSS Statistics 22.0 software (IBM, Armonk, NY, United States).

## Results

### Exercise performance and aerobic capacity

**Figure 2:**
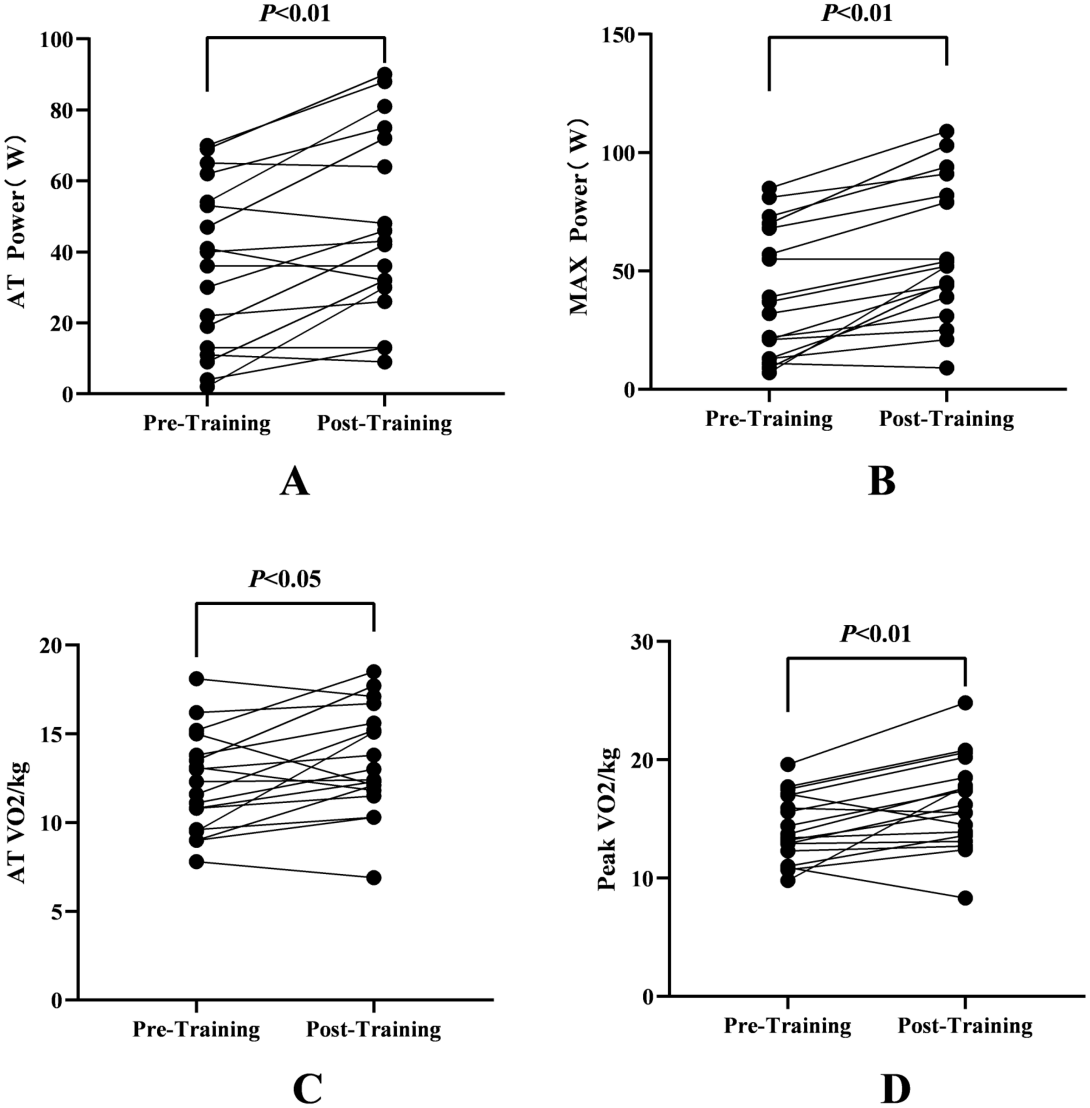
Comparisons in cardiopulmonary fitness pre- and post-training. **(A)** Aerobic threshold (AT) power (W) before and after training. **(B)** Maximal power (MAX Power, W) before and after training. **(C)** Aerobic threshold oxygen consumption (AT VO₂, mL/kg/min) before and after training. **(D)** Peak oxygen consumption (Peak VO₂, mL/kg/min) before and after training.All values are presented as individual data points connected by lines for each participant. Statistical significance is indicated as follows: *p* < 0.05, *p* < 0.01.

Demographic and clinical measures of the participants are shown in Table 1. After 4 weeks of training, Maximal power output (MAX Power) increased significantly from 40.71 ± 27.53 W (pre-training) to 58.71 ± 29.47 W (post-training) (T = −5.84, *p* < 0.001), indicating enhanced anaerobic capacity. Anaerobic threshold power (AT Power) also improved from 36.76 ± 23.6 W to 47.88 ± 26.26 W (T = −3.68, *p* = 0.002), suggesting better endurance performance. Peak oxygen uptake (Peak VO₂/kg) rose from 14.39 ± 2.79 mL/kg/min to 16.46 ± 3.94 mL/kg/min (T = −3.21, *p* = 0.006), demonstrating improved aerobic fitness of PD patients (see Table 2; Figures 2A,B,D).

**Table 1:** Demographic and clinical measures of the participants.

Characteristic	Values
Age	
Mean (SD)	65.2 ± 9.3
Range	35–75
Gender, *n* (%)	
Male	10 (58.8%)
Female	7 (41.2%)
Duration of disease, y	
Mean (SD)	4.4 ± 2.9
Range	0.5–11.0
MMSE	
Mean (SD)	27.2 ± 2.8
Range	24–30
H&Y stage, *n* (%)	
Stage 1	7 (41.2%)
Stage 2	4 (23.5%)
Stage 3	6 (35.3%)
Subtypes of Parkinson’s Disease, *n* (%)	
TD	9 (52.9%)
PIGD	8 (47.1%)
Levodopa equivalent dose (mg), LED	
Mean (SD)	526 ± 204
Range	75–910

TD, tremor-dominant; PIGD, postural instability and gait difficulty.

**Table 2:** Parameter comparisons in cardiopulmonary fitness pre- and post-training.

Variable	Pre-training (*n* = 17)	Post-training (*n* = 17)	Test value	*p*-value
Rest HR (bpm)	84.71 ± 16.29	84.18 ± 15.02	0.26	0.8
AT Power (W)	36.76 ± 23.6	47.88 ± 26.26	−3.68	0.002**
AT VO₂/kg (mL/kg/min)	12.27 ± 2.86	13.54 ± 3.08	−2.37	0.031*
AT HR (bpm)	107 ± 13.3	111.71 ± 13.97	−2.13	0.049*
MAX power (W)	40.71 ± 27.53	58.71 ± 29.47	−5.84	0.000**
Peak VO₂/kg (mL/kg/min)	14.39 ± 2.79	16.46 ± 3.94	−3.21	0.006**
MAX HR (bpm)	112.59 ± 13.76	118.24 ± 14.18	−2.7	0.016*
Recovery HR (bpm)	103.82 ± 13.14	109.65 ± 14.52	−2.48	0.025*
VE/VCO_2_ slope	32.66 ± 5.08	32.02 ± 5.63	0.72	0.828
OUES (mL/min)	1304.27 ± 298.91	1337.94 ± 470.22	−0.53	0.429

MAX Power, maximal power output; AT, anaerobic threshold power output; OUES, oxygen uptake efficiency slope; MAX HR, maximal heart rate; *p < 0.05; **p < 0.01.

### Cardiovascular adaptations

After 4 weeks of training, Maximal heart rate (MAX HR) increased from 112.59 ± 13.76 bpm to 118.24 ± 14.18 bpm (T = −2.7, *p* = 0.016), possibly reflecting enhanced cardiovascular response to exercise. Recovery heart rate (Recovery HR) showed a significant rise from 103.82 ± 13.14 bpm to 109.65 ± 14.52 bpm (T = −2.48, *p* = 0.025), suggesting improved post-exercise cardiac recovery (see Table 2).

### Anaerobic threshold and metabolic efficiency

After 4 weeks of training, AT VO₂/kg increased from 12.27 ± 2.86 mL/kg/min to 13.54 ± 3.08 mL/kg/min (T = −2.37, *p* = 0.031), indicating better metabolic efficiency at submaximal intensities. AT HR also increased from 107 ± 13.3 bpm to 111.71 ± 13.97 bpm (T = −2.13, *p* = 0.049), supporting enhanced endurance capacity (see Table 2; Figure 2C).

### Pulmonary function and other parameters

After 4 weeks of training, Peak expiratory flow (PEF) showed a slight but significant improvement (5.11 ± 1.43 L to 5.17 ± 1.15 L, T = −0.28, *p* = 0.027). However, resting heart rate (Rest HR), ventilatory efficiency (VE/VCO₂ slope), oxygen uptake efficiency slope (OUES), and lung function parameters (FVC, FEV₁, MVV) did not exhibit significant changes (*p* > 0.05), see Table 3 and Figure 2C.

**Table 3:** Parameter comparisons in pulmonary function pre- and post-training.

Variable	Pre-Training (*n* = 17)	Post-Training (*n* = 17)	Test value	*p*-value
FVC (L)	2.64 ± 0.74	2.79 ± 0.74	−2.44	0.481
FEV_1_ (L)	2.23 ± 0.62	2.32 ± 0.62	−1.79	0.605
PEF (L)	5.11 ± 1.43	5.17 ± 1.15	−0.28	0.027*
PEF% (L)	74.34 ± 15.84	76.16 ± 16.68	−0.58	0.093
MVV (L)	78.24 ± 27.24	80.9 ± 27.14	−0.64	0.784

FVC, forced vital capacity; FVC_1_, forced expiratory volume in 1 s; PEF, peak expiratory flow; MVV: maximum voluntary ventilation. *p < 0.05; **p < 0.01.

## Discussion

The present before-and-after study demonstrates that a structured, short-term (4-week), home-based aerobic cycling intervention can elicit significant improvements in key cardiopulmonary parameters among patients with mild-to-moderate Parkinson’s disease. Physical exercise, including aerobic cycling, is an effective intervention for improving motor symptoms and quality of life in PD (9–12). Our findings align with and extend the growing body of evidence supporting the role of aerobic exercise as a vital non-pharmacological strategy in PD management.

The observed significant increases in maximal power output (MAX Power) and peak oxygen uptake (Peak VO₂/kg) are particularly noteworthy. These improvements suggest an enhancement in the overall aerobic capacity and functional reserve of PD patients, which is often compromised due to disease-related deconditioning and physical inactivity (3, 9). The magnitude of improvement in Peak VO₂/kg (~2.1 mL/kg/min) in our short-term intervention is remarkably consistent with the findings of van der Kolk et al. (7), who reported a mean difference of 2.4 mL/kg/min after a 24-week home-based cycling program. This suggests that even a shorter, but supervised and intensive, regimen can initiate substantial physiological adaptations. Furthermore, improved cardiorespiratory fitness has been demonstrated to be associated with increased cerebral blood flow and enhanced cognitive function (10, 23), suggesting that the physiological benefits observed in this study may also have potential positive implications for cognitive impairment in PD patients. The concurrent improvement in anaerobic threshold (AT) measures (power and VO₂) further indicates enhanced metabolic efficiency and endurance at submaximal efforts, which is critically important for performing daily activities without excessive fatigue (8, 12).

The mechanisms underlying these improvements are likely multifaceted (24). Aerobic exercise has been shown to induce neuroplastic changes, including strengthened functional connectivity in the motor cortex (5, 6) and increased release of neurotrophic factors such as Brain-Derived Neurotrophic Factor (BDNF) (13–15). BDNF plays a crucial role in neuronal survival, synaptic plasticity, cardiovascular protective effects, and even mitochondrial biogenesis (16–18, 26). The increase in exercise capacity observed in our study may thus reflect not only peripheral cardiovascular adaptations but also central neuromodulatory effects that improve motor efficiency and reduce the perceived exertion of exercise (11, 12, 19, 27). This is especially relevant in PD, where the central fatigue and dysfunction of the nigrostriatal pathway contribute to reduced exercise tolerance (11, 25).

The significant changes in heart rate parameters—specifically the increase in maximal heart rate (MAX HR) and recovery heart rate (Recovery HR)—point toward a positive effect on cardiovascular autonomic regulation. Autonomic dysfunction, including blunted heart rate response to exercise and impaired recovery, is a common non-motor feature of PD (9, 20). Our results suggest that aerobic exercise may help modulate autonomic nervous system function, improving chronotropic competence and post-exercise recovery. This aligns with emerging research highlighting the potential of exercise to ameliorate autonomic symptoms in early PD (28).

In contrast to the clear cardiovascular benefits, pulmonary function parameters remained largely unchanged except for a slight improvement in Peak Expiratory Flow (PEF). This is consistent with some previous studies that found limited effects of cycling training on static lung volumes in PD patients (21, 22). Respiratory muscle rigidity and bradykinesia may require more targeted respiratory muscle training or longer intervention periods to elicit significant changes. However, the improvement in PEF, which reflects expiratory muscle strength and cough efficacy, is clinically meaningful given the high risk of aspiration pneumonia in PD.

A key strength of our study was the implementation of a supervised home-based model using readily available technology (wearable heart rate monitors and mobile apps). This approach addresses a critical barrier to long-term exercise adherence in PD—accessibility and convenience (7, 8). Remote supervision allowed for real-time monitoring and individualization of exercise intensity, ensuring patients trained within the prescribed aerobic zone (70–80% HRR) while maintaining safety. This model demonstrates the feasibility of delivering high-quality, structured exercise therapy beyond traditional clinical settings, which is essential for scalable and sustainable disease management (7, 12).

One of the main limitations of this study is the absence of a parallel control group. Although a pre-post self-control design was adopted and the assessment conditions and medication adjustments were strictly controlled, the potential effects of the placebo effect or natural disease fluctuations cannot be completely ruled out. Secondly, the small sample size (*n* = 17) and single-center design may limit the generalizability of the results to the broader PD population. In addition, the 4-week intervention duration can only verify short-term effects, and the long-term sustainability of these benefits and their impact on non-motor symptoms (such as cognitive function and quality of life) in PD patients have not been clarified. Future research should adopt a large-sample, multi-center RCT design, set up a blank control or conventional rehabilitation control group, extend the follow-up period to 6–12 months, further verify the long-term effectiveness and safety of the intervention, and explore its potential impact on the progression of PD.

## Conclusion

In conclusion, our findings indicate that a 4-week supervised home-based aerobic cycling program is a feasible and effective intervention for improving cardiopulmonary function in patients with mild-to-moderate Parkinson’s disease. The significant enhancements in aerobic capacity, metabolic efficiency, and cardiovascular response highlight the potential of short-term, technology-assisted exercise programs to mitigate the functional decline associated with PD. This study adds to the compelling evidence base that structured aerobic exercise should be integrated as a cornerstone of comprehensive PD care, with a focus on accessibility and personalization to promote long-term adherence and maximize therapeutic benefits.

## Data Availability

The raw data supporting the conclusions of this article will be made available by the authors, without undue reservation.
